# Robust *Agrobacterium*-Mediated Transient Expression in Two Duckweed Species (Lemnaceae) Directed by Non-replicating, Replicating, and Cell-to-Cell Spreading Vectors

**DOI:** 10.3389/fbioe.2021.761073

**Published:** 2021-11-04

**Authors:** Anton Peterson, Olena Kishchenko, Yuzhen Zhou, Maksym Vasylenko, Anatoli Giritch, Jian Sun, Nikolai Borisjuk, Mykola Kuchuk

**Affiliations:** ^1^ Jiangsu Key Laboratory for Eco-Agricultural Biotechnology Around Hongze Lake, Jiangsu Collaborative Innovation Centre of Regional Modern Agriculture and Environmental Protection, School of Life Sciences, Huaiyin Normal University, Huai’an, China; ^2^ Institute of Cell Biology and Genetic Engineering, National Academy of Science of Ukraine, Kyiv, Ukraine; ^3^ Nomad Bioscience GmbH, Halle, Germany; ^4^ Jiangsu Key Laboratory of Phylogenomics and Comparative Genomics, School of Life Sciences, Jiangsu Normal University, Xuzhou, China

**Keywords:** transient expression, recombinant proteins, expression vector, duckweed, plant expression system

## Abstract

Plant-based transient expression systems have recognized potential for use as rapid and cost-effective alternatives to expression systems based on bacteria, yeast, insect, or mammalian cells. The free-floating aquatic plants of the Lemnaceae family (duckweed) have compact architecture and can be vegetatively propagated on low-cost nutrient solutions in aseptic conditions. These features provide an economically feasible opportunity for duckweed-based production of high-value products via transient expression of recombinant products in fully contained, controlled, aseptic and bio-safe conditions in accordance with the requirements for pharmaceutical manufacturing and environmental biosafety. Here, we demonstrated *Agrobacterium*-mediated high-yield transient expression of a reporter green fluorescent protein using deconstructed vectors based on potato virus X and sweet potato leaf curl virus, as well as conventional binary vectors, in two representatives of the Lemnaceae (*Spirodela polyrhiza* and *Landoltia punctata*). Aseptically cultivated duckweed populations yielded reporter protein accumulation of >1 mg/g fresh biomass, when the protein was expressed from a deconstructed potato virus X-based vector, which is capable of replication and cell-to-cell movement of the replicons in duckweed. The expression efficiency demonstrated here places duckweed among the most efficient host organisms for plant-based transient expression systems, with the additional benefits of easy scale-up and full containment.

## Introduction

Plant-based transient expression systems are widely used for studying gene and protein functions, metabolic engineering, and biosynthesis of valuable recombinant products (Reed et al., 2017; Tusé et al., 2020). Second-generation plant virus-based vectors (or deconstructed viral vectors) in combination with *Agrobacterium*-mediated delivery provide fast and scalable expression of recombinant DNA in dicotyledonous plants such as *Nicotiana benthamiana* or *Lactuca sativa* (Peyret and Lomonossoff, 2015; Yamamoto et al., 2018; Shanmugaraj et al., 2020). RNA and DNA viruses have been used to generate plant expression vectors (Marillonnet et al., 2005; Lindbo, 2007; Chen et al., 2011; Peyret and Lomonossoff, 2013; Yu et al., 2020; Torti et al., 2021). Replicons derived from these vectors can self-amplify in host cells to increase their copy number and persistence, and may be capable of cell-to-cell movement, which substantially increases the number of cells expressing the target products. The most prominent examples of such vectors are the magnICON vectors based on turnip vein-clearing virus (TVCV), crucifer-infecting tobacco mosaic virus (cr-TMV), or potato virus X (PVX) (Marillonnet at al., 2004; Torti et al., 2021).

PVX belongs to the Potexvirus genus of the Alphaflexiviridae family and has a positive single-stranded RNA genome. The PVX genome includes genes encoding RNA-dependent RNA polymerase and coat protein (CP), as well as three partially overlapping genes, or a triple gene block encoding three movement proteins (MPs) (Atabekov et al., 2007). The PVX-based deconstructed vectors that encode MPs and modified CPs are replicating vectors that can move from cell to cell and systematically. These vectors are used for *Agrobacterium*-mediated transient expression in a number of dicotyledonous plant species (Giritch et al., 2006; Larsen and Curtis, 2012; Schulz et al., 2015; Mardanova et al., 2017; Torti et al., 2021). Geminiviruses are also often used for deconstructed viral systems (Chen et al., 2011; Baltes et al., 2014). Monopartite sweet potato leaf curl virus (SPLCV) belongs to the Begomovirus genus of the Geminiviridae family, which comprises twin-spherical viruses with single-stranded circular DNA genomes. Begomoviruses replicate through double-stranded DNA intermediates using a rolling-circle mechanism. Their replication is directed by two intergenic regions and replication-associated proteins (AC1, AC2, AC3, and AC4) that are encoded by the virus genome. Whiteflies *(Bemisia tabaci)* exclusively transmit the Begomoviruses, and these viruses infect only dicotyledonous plants (Bi and Zhand, 2012). Geminivirus-based deconstructed vectors are replicating vectors but do not undergo cell-to-cell movement.

Recent improvements in deconstructed viral vector elements have increased yields of recombinant products and expanded the list of terrestrial plant species that can serve as host organisms for high-yield transient expression (Diamos et al., 2016; Yamamoto et al., 2018; Diamos and Mason, 2019; Meshcheriakova and Lomonossoff, 2019; Peyret et al., 2019; Diamos et al., 2020; Torti et al., 2021). Using whole terrestrial plants as host organisms substantially complicates the certification of production sites in accordance with the pharmaceutical manufacturing and biosafety regulatory requirements to ensure full containment. Expanding transient expression methods to plants that can be easily and economically cultivated in fully contained conditions will thus provide substantial scientific and industrial benefits.

Lemnaceae (duckweed) is a family of 37 free-floating aquatic monocotyledonous plant species (Tippery and Les, 2020), with *Spirodela polyrhiza* being widely distributed around the world and *Landoltia punctata* being one of the most common duckweed species in tropical and sub-tropical areas. Both species have high protein contents (20–25% of dry biomass) and fast growth rates achieved through clonal reproduction (biomass doubling in 48 h) under optimal cultivation conditions. Considering the well-recognized benefits of these plants as a platform for production of pharmaceutical proteins (Gasdaska et al., 2003; Stomp, 2005; Everett et al., 2012; Yang et al., 2021), Lemnaceae species are promising host organisms for developing efficient transient expression systems that will be compatible with fully contained cultivation.

Here, we demonstrate a high-yield transient expression system using two duckweed species along with various vectors (non-replicating, replicating, and cell-to-cell spreading) previously used for transient expression in terrestrial dicotyledonous plants, delivered through a simple, cheap, and easily scalable *Agrobacterium*-mediated method.

## Materials and Methods

### Plant Materials

Duckweed samples were collected from small ponds in Ukraine: *Spirodela polyrhiza* (N 50″673456, E 30″716611) and China: *Landoltia punctata* (N 33″599292, E 119″05674). Following collection, the duckweed samples were surface sterilized in 0.5% sodium hypochlorite and washed with autoclaved water. The procedure was repeated 2 days later, and fronds were placed on solid SH nutrient medium for propagation (Schenk and Hildebrandt, 1972). The identity of each species was confirmed by double barcoding using primers specific for the chloroplast DNA intergenic spacers *atpF-atpH* (ATP) and *psbK-psbL* (PSB) (Borisjuk et al., 2015).

**FIGURE 1:**
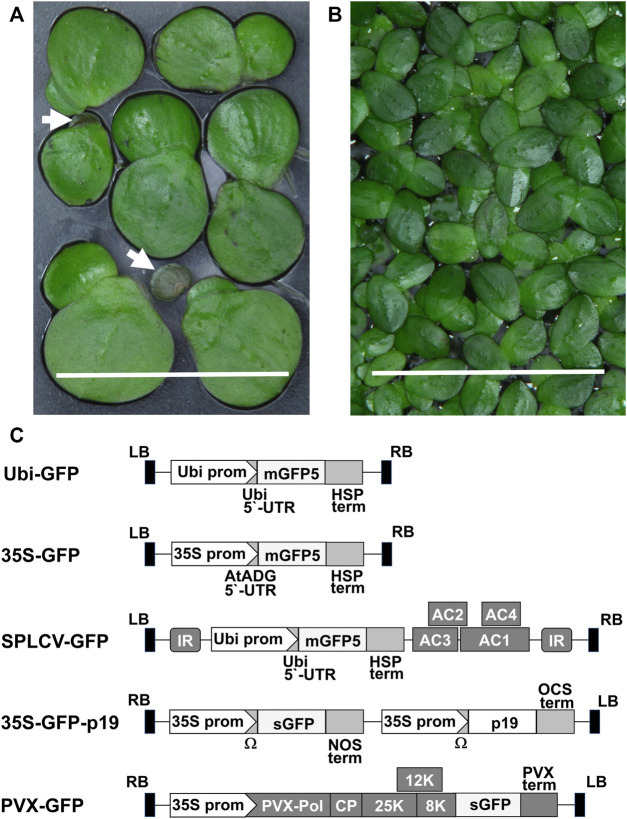
Duckweed species and vector constructs used in research: **(A)** *Spirodela polyrhiza* fronds and turions (white arrows show turions), bar = 1 cm. **(B)** *Landoltia punctata* fronds, bar = 1 cm. **(C)** Schematic representation of the T-DNA of vectors: Ubi prom–the promoter of the *N. tabacum Ubiquitin4 U4* gene, mGFP5—coding sequence of modified GFP, sGFP–coding sequence of synthetic GFP, HSP term–the transcription terminator of the *A. thaliana HEAT SHOCK PROTEIN* 18.2 gene, *35S* prom–the сauliflower mosaic virus *35S* promoter, AtADH 5′-UTR–translational enhancer from the 5′-UTR of the *A. thaliana ALCOHOL DEHYDROGENASE* gene, AC1, AC2, AC3 and AC4–coding regions of SPLCV replication-associated proteins, IR - SPLCV intergenic region, p19—the tomato bushy stunt virus suppressor of gene silencing, Ω–the tobacco mosaic virus translational enhancer of 5′-leader sequence, NOS term–the transcription terminator of the *A. tumefacience NOPALINE SYNTHASE* gene, OCS term–the transcription terminator of the *A. tumefacience OCTOPINE SYNTHASE* gene, 8, 12, 25 K–PVX triple gene block movement proteins, PVX term–the PVX transcription terminator, CP–coding sequence of the PVX coat protein, PVX-pol–PVX RNA-dependent RNA polymerase gene, LB, RB–left and right borders of the T-DNA.

The duckweed plants used for the experiments (Figures 1A,B) were aseptically cultivated in 500-ml flasks containing 200 ml liquid SH medium with 5 g/L sucrose in an incubator at 23 ± 1°C with a photon flux density of 100–120 μmol m^−2^ s^−1^ provided by cool white fluorescent bulbs in a 16 h light/8-h dark cycle for 1 month. Descriptions of reference plants are provided in Supplementary Method S1.

### Expression Vectors and Agrobacteria Strain

A schematic representation of the vectors used for expression of the green fluorescent protein (GFP) reporter in plants is shown in Figure 1C. Vectors PVX-GFP and 35 S-GFP-p19 were generously donated by Nomad Bioscience GmbH (Halle/Saale, Germany) to the Institute of Cell Biology and Genetic Engineering, National Academy of Science of Ukraine, Kyiv, Ukraine.

Vectors SPLCV-GFP, Ubi-GFP, and 35 S-GFP were kindly provided to the Jiangsu Key Laboratory for EcoAgricultural Biotechnology around Hongze Lake, School of Life Sciences, Huaiyin Normal University, Huai’an, China by Prof. Jian Sun at the Jiangsu Key Laboratory of Phylogenomics and Comparative Genomics, School of Life Sciences, Jiangsu Normal University, Xuzhou, Jiangsu, China.

The non-replicating vector 35S-GFP contains an *mGFP5* variant fused with 6His-tag, expressed under the control of the 35S promoter of cauliflower mosaic virus (CaMV) and a translational enhancer from the 5′-UTR of the *Arabidopsis thaliana* *ALCOHOL DEHYDROGENASE (ADH)* gene. In the non-replicating vector Ubi-GFP, expression of *mGFP5* is controlled by the promoter and 5′-UTR of the *Nicotiana tabacum Ubiquitin4 (U4)* gene (Yu et al., 2020). The replicating vector SPLCV-GFP contains a transcription unit identical to that of Ubi-GFP, coupled with the coding region of SPLCV replication-associated proteins (AC1, AC2, AC3, and AC4) and two intergenic regions (IRs), which provide replication (Yu et al., 2020).

The non-replicating vector 35S-GFP-p19 (Giritch et al., 2017) carries the coding sequences of the *sGFP* variant and the p19 suppressor of gene silencing from tomato bushy stunt virus, both under the control of the *35S* promoter and the translational enhancer *Ω* 5′-leader sequence from tobacco mosaic virus. The PVX-based vector PVX-GFP (Marillonnet et al., 2012; Torti et al., 2019) contains sequences encoding RNA-dependent RNA polymerase, coat protein, and movement proteins, providing replication and cell-to-cell movement of vector-derived replicons.

The five binary vectors were transferred to the *Agrobacterium* tumefaciens strain GV3101 (Nguyen et al., 2012; Park et al., 2014). Agrobacteria were grown on LB medium supplemented with antibiotics (50 mg/L rifampicin, 25 mg/L gentamicin, 50 mg/L spectinomycin, and 100 mg/L kanamycin).

### Infiltration of Plant Materials by *Agrobacterium* Suspensions

Agrobacteria were cultivated in 0.5 L LB medium supplied with the appropriate antibiotics in 1.5-L hermetic vessels with intensive mixing by magnetic stirrers at ∼700 rpm for 48 h at 28°C. The accumulated Agrobacteria were collected by centrifugation at 4,000 *g* for 10 min at room temperature and the cells were re-suspended in the same volume of 10 mM Mg-MES buffer (Marillonnet et al., 2005). To submerge the duckweed plants, which usually float on the surface of the water, the duckweed fronds were packed into autoclaved reticulated stainless-steel containers (tea infusers) and then immersed in the *Agrobacterium* suspensions. Plants were infiltrated with the *Agrobacterium* suspension in a vacuum chamber with the maximum vacuum (below –0.1 MPa).

Following infiltration, *S. polyrhiza* and *L. punctata* fronds were briefly washed with autoclaved water, blotted on filter paper in Petri dishes, and cultivated at 22°C. After 24 h, plant material was transferred to liquid or solid SH medium supplemented with sucrose (30 g/L) and timentin (600 mg/L) to eliminate the *Agrobacterium* growth. For further incubation, duckweeds were transferred to a climate chamber with a 16 h/8 h light/dark photoperiod at 18°C for the light period and 16°C for the dark period.

Maintenance of the *Lactuca sativa* and *Nicotiana benthamiana* reference plants after infiltration is described in Supplementary Method S2.

### Visual Monitoring and Photo-Documentation of Green Fluorescent Protein-specific Fluorescence *in Planta*

Visual monitoring of GFP fluorescence in duckweed fronds, *L. sativa*, and *N. benthamiana* tissues was performed every day to define the phase of maximal reporter protein accumulation and determined the appropriate time for protein extraction. The plant material for protein extraction was collected at the stage when no further increase of specific fluorescence was observed.

The visual observations of GFP fluorescence dynamics in duckweed fronds after agroinfiltration were performed during cultivation in aseptic conditions. For the non-replicating vector 35S-GFP-p19 in *S. polyrhiza*, and the 35S-GFP and Ubi-GFP vectors in *L. punctata*, the monitoring was usually performed during the 30 days post infiltration (dpi). For the replicating vector SPLCV-GFP (in *L. punctata*), the monitoring was performed until 90 dpi. For the replicating and cell-to-cell spreading vector PVX-GFP (in *S. polyrhiza*), the monitoring was performed for 1 year post infiltration.

The monochromatic light sources, long wave pass light filters, and eyepieces, as well as the photocameras and software used for image processing, are described in Supplementary Method S3.

### Measurement of Green Fluorescent Protein Accumulation in Plant Tissue

The accumulation of GFP reporter protein in plant biomass was measured following soluble protein extraction and separation by gel electrophoresis (SDS-PAGE, 7.5% gel). This procedure eliminates plant pigments and fluorophores, and identifies GFP by its specific fluorescence and electrophoretic mobility. Fronds (5–10 g) aseptically grown in liquid nutrient medium were collected (without any frond preselection) at the phase of maximum fluorescence for each vector, and used for protein extraction. Material preparation from reference plants is described in Supplementary Methods.

For protein extraction, plant material was ground in a kitchen blender (17,500 rpm, on ice) in 8 M urea solution containing 0.1 mM PMSF and 0.1 mM EDTA and centrifuged for 15 min at 12,000 g, 4°C. Supernatants were used for further analysis. To preserve the fluorescent properties of GFP, the heat denaturation step routinely used in sample preparation for SDS-PAGE separation was omitted. After SDS-PAGE protein fractionation, thin strips of gel containing specific fluorescent bands were cut out (in the dark, using an appropriate laser for GFP fluorescence excitation), and the reporter proteins were eluted from the gel strips.

GFP elution was performed in 12-ml flat-bottom glass vials (orbital shaker, 72 h, 4°C) using the gel running buffer solution (1x Tris-glycine buffer solution for SDS-PAGE) containing 0.1 mM PMSF and 0.1 mM EDTA. Fluorescence measurements of the solutions were performed in wells of flat-bottom 96 micro-well plates (Corning) using Infinite M200 PRO microplate reader (TECAN, Switzerland) or in glass cuvettes with 10-mm optical path using FLUORAT-02-Panorama spectrofluorometer (Lumex Instruments, RF). For GFP fluorescence measurements, 395 nm excitation and 510 nm emission wavelengths were used for sGFP and 479 nm excitation/512 nm emission wavelengths were used for mGFP5. The reporter protein contents in plant biomass were calculated using calibration chart trend line equations, and dilution coefficients, which were calculated based on the volumes used in the grinding, extraction, SDS-PAGE, and elution.

The calibration charts to convert fluorescence into concentration were built for both sGFP and GFP5 using dilutions of purified proteins expressed in *E. coli* (for details see Supplementary Method S4). All measurements of the bacteria-expressed sGFP and mGFP5 were identical to the measurements of plant-expressed protein described above. The trend line equations in the calibration charts were generated using LibreOffice Calc free software.

### Statistical Analysis

A minimum of three independent experiments were conducted for each experimental condition, with a minimum of three technical repeats of measurements for each independent experiment. For each experimental group of independent experiments, mean values and standard deviations were calculated using LibreOffice Calc free software. Representative photos were chosen among a minimum of six photos of transparent containers/Petri dishes with duckweed.

The normality of data distribution in each group (Kolmogorov-Smirnov test and Shapiro-Wilk test) and the homogeneity of variances (Levene tests based on mean, median, median and with adjusted df, trimmed mean) were tested. Based on the results of Levene median and median with adjusted df. tests, One-Way ANOVA (Tukey Post Hoc multiple comparison) was selected for estimation of the significance of differences. “IBM SPSS Statistic” (Version 28.0.0.0) software was applied for the calculation. All the calculations were performed for *α* = 0.05.

## Results

### *Agrobacterium*-Mediated Delivery of Vectors and Dynamics of Reporter Protein Fluorescence

To test the possibility of high-efficiency transient expression in duckweed (*S. polyrhiza* and *L. punctata*), we used a range of expression vectors carrying a reporter gene encoding GFP. The nonreplicating vectors Ubi-GFP and 35S-GFP and the replicating vector SPLCV-GFP were tested in *L. punctata*. The non-replicating vector 35S-GFP-p19 and the replicating vector PVX-GFP, with the capability of cell-to-cell movement, were tested in *S. polyrhiza*. All vectors were delivered into the plants by *Agrobacterium* through vacuum infiltration.

Reference plants were used in each experimental cycle to confirm the activity of the *Agrobacterium* cultures prepared for infiltration (Supplementary Data S1, Supplementary Figure S1). *N. benthamiana*, a well-documented host plant for transient expression, was used as a positive control (or reference) for 35S-GFP-p19 and PVX-GFP vectors, and *L. sativa* was used as a positive control for the Ubi-GFP, 35S-GFP, and SPLCV-GFP vectors. After each vacuum infiltration cycle, aseptically cultivated *S. polyrhiza* and *L. punctata*, and reference *N. benthamiana* plants, were completely vitrified. Reference *L. sativa* plants usually had a lower level of infiltration: the basal parts of the leaves (approximately 1/5–1/4 of the leaf surface) did not appear vitrified.

We monitored the dynamics of GFP expression by naked-eye observations of the total GFP specific fluorescence from the infiltrated duckweed populations, which were aseptically cultivated in liquid SH nutrition medium, supplied with timentin to eliminate the *Agrobacterium*. To trace in detail the dynamics of the brightness of fluorescence on surfaces of the infiltrated duckweed fronds, we transferred the fronds to SH solid medium with timentin. The time-course of GFP accumulation determined by the dynamics of brightness and size of the fluorescent zones on frond surfaces generally could be divided in three phases: increasing fluorescence, stationary phase of maximum fluorescence, and decreasing fluorescence, with some variation depending on the vector type.

#### Green Fluorescent Protein Expression From the Non-replicating Vectors

The first signs of GFP expression visible by the naked eye in *N. benthamiana* or *L. sativa* Were detected at 2 dpi for all tested non-replicating vectors. In the infiltrated duckweed, the first visual sign of GFP fluorescence occurred at 3 to 4 dpi for the 35S-GFP vector in *L. punctata* and for the 35S-GFP-p19 vector in *S. polyrhiza*. There were no visible signs of GFP-specific fluorescence in *L. punctata* infiltrated with *Agrobacterium* containing the Ubi-GFP vector during the whole period of observation (30 days).

**FIGURE 2:**
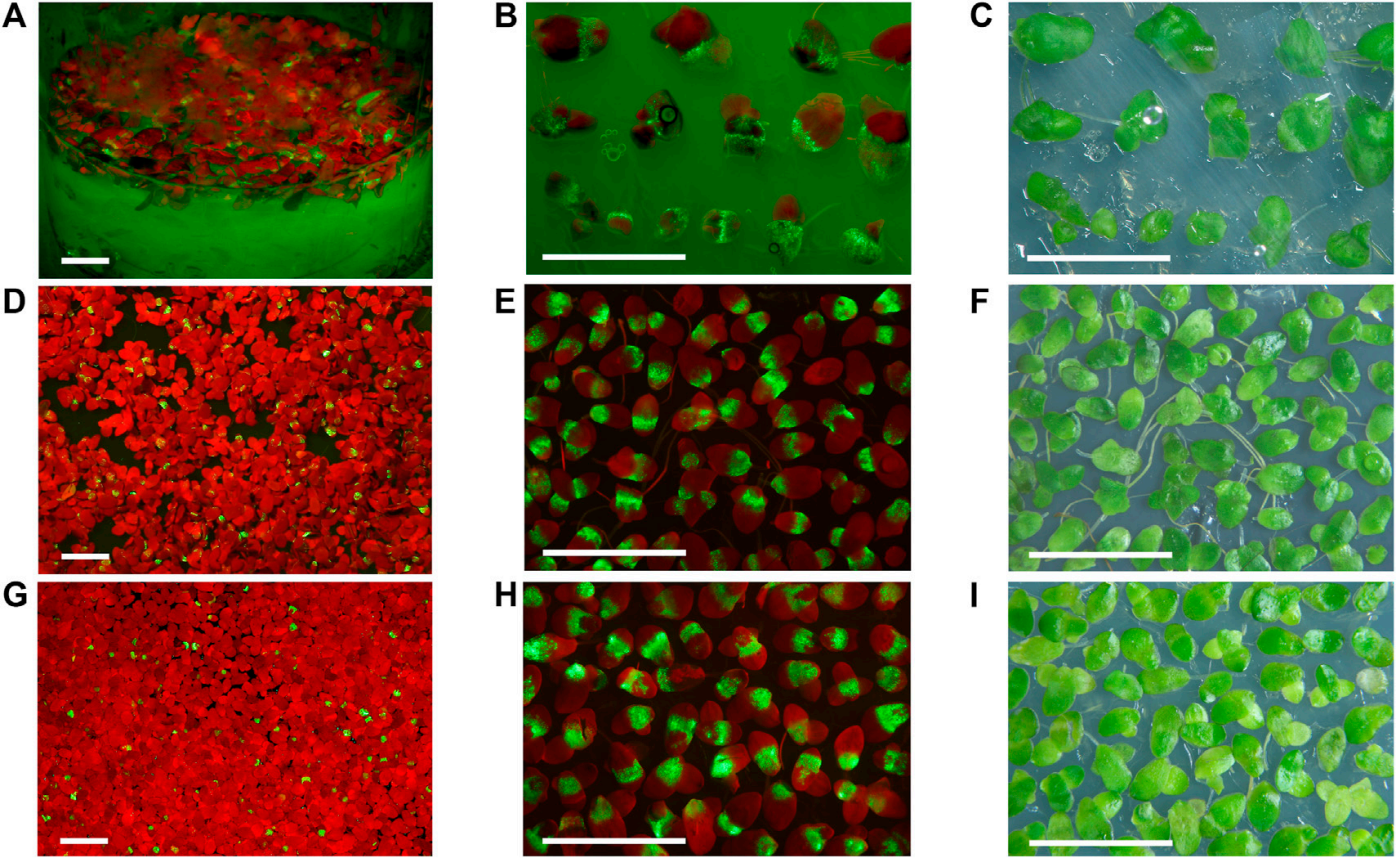
Duckweed transiently expressing GFP from the non-replicating vectors 35S-GFP-p19 and 35S-GFP and the replicating vector SPLCV-GFP. **(A,D,G)** The appearance of duckweed populations infiltrated with *Agrobacterium* bearing the 35S-GFP-p19 **(A)**, 35S-GFP **(D)**, and SPLCV-GFP vectors **(G)**. **(B,C)** Selected fronds expressing GFP from *S. polyrhiza* population infiltrated with *Agrobacterium* bearing the 35S-GFP-p19 vector, 10 dpi. **(E,F)** Selected fronds expressing GFP from *L. punctata* population infiltrated with *Agrobacterium* bearing the 35S-GFP vector, 10 dpi. **(H,I)** Selected fronds expressing GFP from *L. punctata* population infiltrated with *Agrobacterium* bearing the SPLCV-GFP vector, 30 dpi. **(C,F,I)** Images were taken under artificial white light. **(A,B)** Images were taken under monochrome excitation light (400 nm) through a long wave pass light filter (450 nm). **(D,E,G,H)** Images were taken under monochrome excitation light (488 nm) through a long wave pass light filter (520 nm). Bar = 1 cm.

For the 35S-GFP and 35S-GFP-p19 vectors, the phase of increasing GFP fluorescence in duckweed usually occurred 1 week after the first visible sign of fluorescence. The appearance of new fluorescent spots on the surface of infiltrated fronds usually stopped at 7 or 8 dpi. The brightness of GFP fluorescence started to decrease at 11 or 13 dpi and completely disappeared around 27–30 dpi. There were no signs of necrosis in fronds over-expressing GFP (Figures 2A–F).

#### Green Fluorescent Protein Expression From the Replicating Vector

In *L. sativa* infiltrated with *Agrobacterium* bearing the SPLCV-GFP vector, we detected the first signs of GFP expression visible by the naked eye at 2 dpi. By contrast, we detected GFP fluorescence in *L. punctata* starting at 5 to 6 dpi. New fluorescent spots on frond surfaces infiltrated with *Agrobacterium* bearing the SPLCV-GFP vector (which cannot move cell to cell) appeared up to 1 month after infiltration. The stationary phase of maximum fluorescence in *L. punctata* was observed between 30 and 40 dpi and the brightness of GFP fluorescence usually started to decrease from 40 to 43 dpi, with fluorescence completely disappearing at 80–90 dpi (Figures 2G–I).

#### Green Fluorescent Protein Expression From the Replicating and Cell-To-Cell Spreading Vector

**FIGURE 3:**
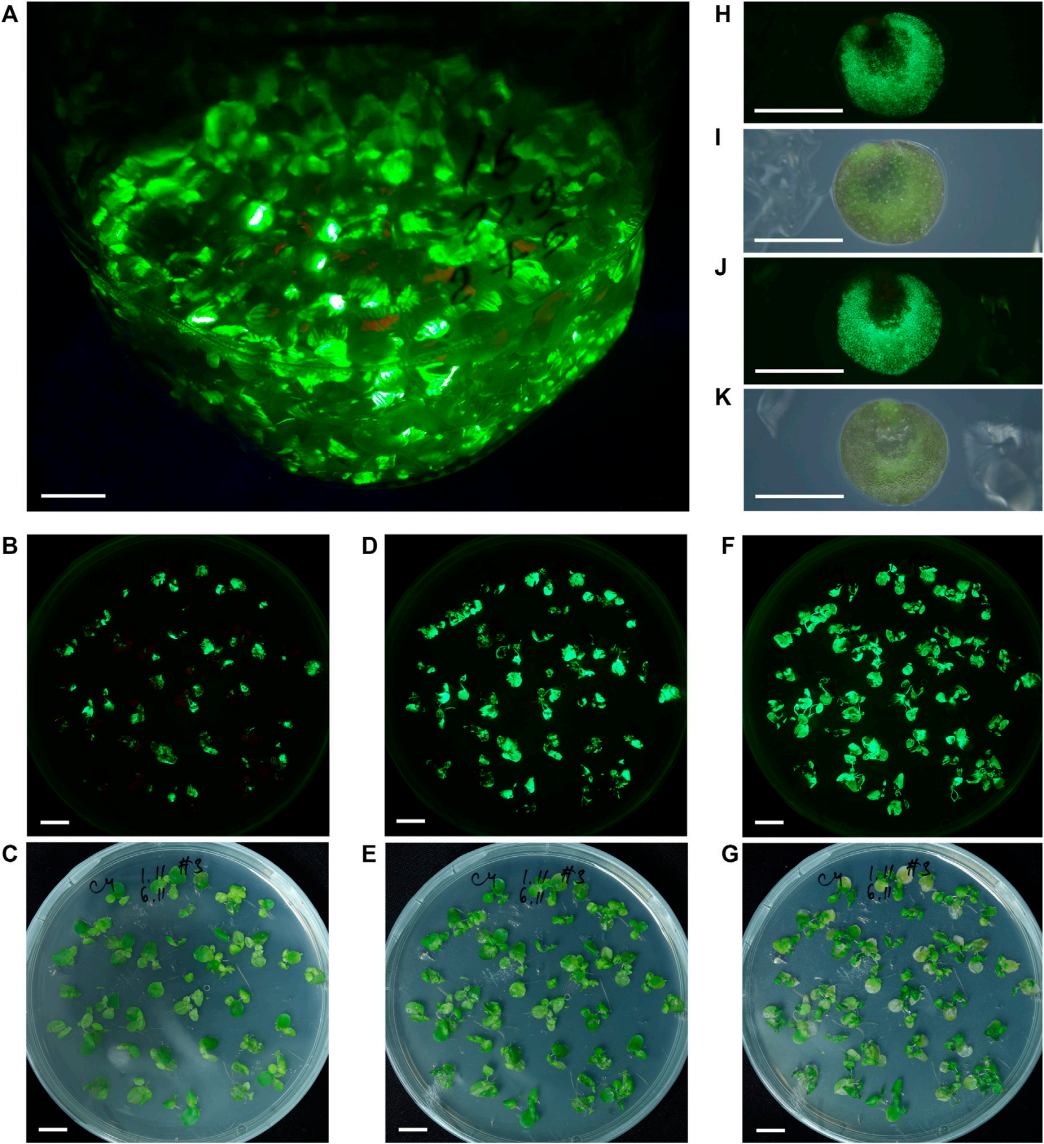
*S. polyrhiza* fronds and turions transiently expressing GFP from the PVX-GFP vector. **(A)** The appearance of fronds population maintained in liquid medium after agro-infiltration, 30 dpi. **(B)–(G)** The dynamics of GFP fluorescence in agro-infiltrated fronds maintained on solid medium at 8 dpi **(B,C)**, 11 dpi **(D,E)**, and 20 dpi **(F,G)**. **(H,I)** Air-facing side of the turion. **(J,K)** Waterfacing side of the turion. **(A,B,D,F,H,J)** Images were taken under monochrome excitation light (400 nm) through a long wave pass light filter (450 nm). **(C,E,G,I,K)** Images were taken under artificial white light. **(A–G)** Bar = 1 cm, **(H–K)** Bar = 1 mm.

In *S. polyrhiza* frond populations infiltrated with Agrobacterium bearing the PVX-GFP vector and grown in liquid medium, we continuously observed the generation of new daughter fronds with fluorescent spots followed by the expansion of the area of fluorescence on the frond surface. During the second and third weeks after infiltration, the ratio of fluorescing to non-fluorescing fronds continuously increased, usually reaching >50%. The period of maximal GFP fluorescence usually started at 20–25 dpi and lasted up to 40–50 dpi (Figure 3A). The overexpression of GFP was accompanied by the appearance of zones of necrosis and accelerated death of fronds.

Cultivation of fronds infiltrated with *Agrobacterium* bearing the PVX-GFP vector on solid medium allowed us to trace in detail the fluorescence dynamics on frond surfaces, which resulted from cell-to-cell or frond-to-frond spreading of the vector transcript. The dynamics of fluorescence of *S. polyrhiza* fronds grown on solid nutrition medium during the phase of increasing fluorescence is shown in Figures 3B–G, Supplementary Figure S2. The main difference between the transient expression dynamics on liquid and solid nutrition medium was the efficiency of cell-to-cell spreading and the resulting level of frond involvement: on solid medium the transient expression could reach almost 100% involvement of duckweed fronds and their surfaces.

We continued to monitor GFP-specific fluorescence in the *S. polyrhiza* cultures infiltrated with *Agrobacterium* bearing the PVX-GFP vector and maintained in aseptic cultivation on liquid nutrition medium for over 1 year after infiltration, and found that in a small portion of fronds, the bright GFP-specific fluorescence zones were still present after 1 year. After several months of cultivation occasionally *S. polyrhiza* fronds infiltrated with the PVX-GFP vector formed turions (as depicted in Figure 1A), some of which had surfaces with GFP-specific fluorescence (Figures 3H–K).

### Accumulation of the Reporter Protein in Duckweed Biomass

To assess the prospects of using duckweed as a potential host for transient expression of various recombinant products, we estimated the average amount of the GFP reporter protein in a bulk sample of infiltrated duckweed biomass. No GFP fluorescence was observed with the non-replicating vector Ubi-GFP in *L. punctata*. Extracts from duckweed infiltrated with *Agrobacterium* bearing the SPLCV-GFP, 35S-GFP, or 35S-GFP-p19 vectors showed a slight GFP-specific fluorescent band on unstained SDS-PAGE gels (Supplementary Figure S3) but no corresponding visible protein bands were observed on Coomassie stained gels.

**FIGURE 4:**
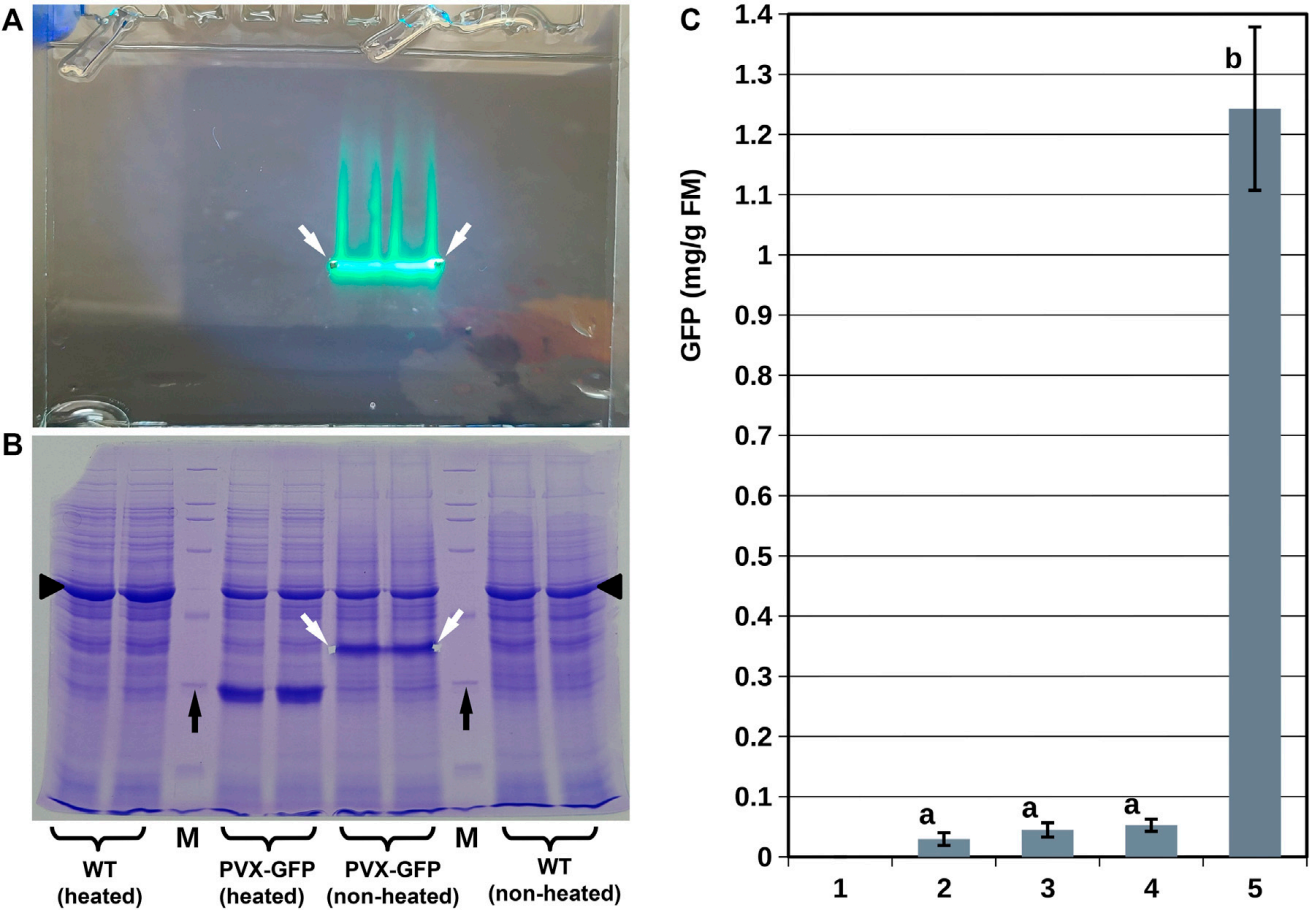
Analysis of GFP accumulation in duckweed biomass. **(A,B)** SDS-PAGE analysis of protein extracts from *S. polyrhiza* infiltrated with *Agrobacterium* bearing the PVX-GFP vector. **(A)** Representative image of the gel before staining; image was taken using artificial white light combined with light from a 365 nm LED without any long wave pass light filters. **(B)** Image of the gel shown in **(A)** after staining with Coomassie dye. **(C)** GFP accumulation in duckweed population infiltrated with *Agrobacterium* bearing different vectors (duckweed/vector): **1–** *L. punctata*/Ubi-GFP vector (no accumulation), 10–12 dpi; **2** - *L. punctata*/35S-GFP vector, 10–12 dpi; **3–** *L. punctata*/SPLCV-GFP vector, 25–30 dpi; **4–** *S. polyrhiza*/35S-GFP-p19 vector, 10–12 dpi; **5–** *S. polyrhiza*/PVX-GFP vector, 20–25 dpi. White arrows show gel notches marking the positions of the GFP fluorescence bands; black arrows show the positions of the 29 kDa bands of the protein molecular weight marker; black triangles indicate the position of large subunit RuBisCO. M—protein molecular weight marker. WT—extracts from *S. polyrhiza* not infiltrated by *Agrobacterium*. Error bars indicate doubled standard deviations (*n* = 3). Values with different letters are significantly different (Tukey Post Hoc multiple comparison, *p* < 0.05).

By contrast, the protein extracts from *S. polyrhiza* infiltrated with *Agrobacterium* bearing the PVX-GFP vector showed prominent GFP-specific fluorescent bands on unstained gels, which corresponded to major bands on Coomassie-stained gels (Figures 4A,B). We confirmed that the molecular weight of the protein in these bands corresponded to that of GFP (∼27 kDa) by running the same extracts after they had been completely denatured (by heating) in parallel with the unheated extracts. It is worth noting that the high GFP accumulation in *S. polyrhiza* infiltrated with *Agrobacterium* bearing the PVX-GFP vector coincided with slight suppression of the major plant photosynthetic protein RuBisCO, in a manner similar to that described for *N. benthamiana* (Marillonnet et al., 2004).

The quantification of the GFP translated into 0.02–0.06 mg/g fresh mass (FM) for the SPLCV-GFP, 35S-GFP, and 35S-GFP-p19 vectors and 1.24 ± 0.13 mg/g for the PVX-GFP vector (Figure 4C). In *N. benthamiana*, the PVX-GFP vector resulted in an average GFP accumulation of 4.3 ± 1.1 mg/g fresh mass. In *L. sativa*, the SPLCV-GFP vector resulted in an average GFP accumulation of 0.85 ± 0.21 mg/g fresh mass (Supplementary Figure S4).

## Discussion

Efficient methods of stable genetic transformation have been developed for some duckweed species (reviewed in Yang et al., 2020). Genetically modified duckweed has been used for different applications, including high-yield expression of various recombinant proteins (Gasdaska et al., 2003; Cox et al., 2006; Vunsh et al., 2007; Yang et al., 2021). These lines of evidence suggest that duckweed can tolerate high levels of recombinant protein accumulation. High levels of GFP in duckweed cells following transient expression of GFP were previously demonstrated by biolistic and *Agrobacterium*-mediated methods using duckweed callus-like cluster structures (Cantó-Pastor et al., 2015; Khvatkov et al., 2015). Here, we demonstrated *Agrobacterium*-mediated high-yield transient expression of the GFP reporter protein in duckweed fronds. The recombinant protein accumulation using the PVX-GFP vector favorably compares to the highest yields reached in transgenic duckweed, as reviewed by Yang et al. (2021). Based on simple calculations, our data for GFP accumulation of 1.2 mg/g fresh biomass converts to ∼24 g/kg of dry biomass, considering ∼5% dry biomass in fresh biomass of *S. polyrhiza* measured in our experiments.

We tested three different types of expression vectors: non-replicating, replicating, and replicating with the ability to spread cell-to-cell; these vectors are primarily used in dicotyledonous species. Two non-replicating vectors 35S-GFP and 35S-GFP-p19, as well as the replicating SPLCV-GFP vector, provided approximately equal efficiency of transient GFP expression, which resulted in fluorescence that was visible to the naked eye in duckweed fronds. The non-replicating Ubi-GFP vector did not demonstrate detectable accumulation of reporter protein in duckweed, probably because the *Ubi* promoter of the *N. tabacum U4* gene is weak in *L. punctata*. Therefore, we concluded that the replicating elements of SPLCV are functional in the monocotyledonous duckweed *L. punctata*.

The substantial accumulation of GFP in *S. polyrhiza* fronds directed by the PVX-GFP vector led to their accelerated death, but moving the vector-derived replicons to newly developed fronds resulted in the continuous persistence (at least during the 1-year period of observation) of the replicons and long-lasting GFP expression in the infiltrated duckweed population. The long-term, bright GFP fluorescence in *S. polyrhiza* population clearly demonstrated high activity and efficiency of the viral proteins encoded by the PVX-GFP vector in duckweed cells, which also ensures systemic spreading of the recombinant replicons from maternal fronds to daughter fronds during vegetative propagation. The GFP signal spread, especially in a vascular system (Supplementary Figures S2B,F,J) is indicative for virus movement.

Another evidence of the efficiency of the PVX proteins associated with cell-to-cell and systemic movement is our observation of GFP expression in turions. The *S. polyrhiza* turions resemble potato tubers in their formation, dormancy, and germination, playing a similar role in nutrient storage and clonal propagation (Appenroth et al., 1996). These findings reveal that the PVX-GFP vector transcripts may spread systemically not only from mother fronds to daughter fronds, but also from mother fronds to the storage organs, the turions. Thus, the deconstructed vector based on PVX, which infects terrestrial dicotyledonous plants, is also highly efficient in the monocotyledonous aquatic duckweed.

The number of cells in duckweed frond tissue that are competent to receive the T-DNA of The vectors from *Agrobacterium*, as well as the number of fronds having such cells in duckweed populations, seem to be the main limiting factors for efficient accumulation of the targeted protein in duckweed. Additionally, when using the non-replicating and replicating vectors, the high vegetative multiplication rate of duckweed fronds results in rapid “dilution” of the expressing fronds, among newly developed (after infiltration) non-expressing fronds, which decreases the ratio of target product to biomass. The ability of the PVX-based vector transcripts to move cell-to-cell in fronds and to spread systemically during vegetative propagation overcame these limitations, leading to 50% involvement in transient expression of infiltrated duckweed population cultivated on liquid medium and up to 100% when cultivated on solid medium.

By performing all stages of the transient expression process in duckweed in aseptic conditions, we demonstrated that Lemnaceae species can be turned into an efficient plant bioreactor for high-yield transient expression in full containment. Accumulation of target recombinant proteins in plant biomass using deconstructed expression vectors could reach up to 5 mg/g of *N. benthamiana* fresh mass (Marillonnet et al., 2004; Lindbo, 2007). The reporter protein accumulation of >1 mg/g fresh mass that we obtained in *S. polyrhiza* using the PVX-based vector demonstrates the potential of a duckweedbased expression system as an alternative to *N. benthamiana*-based systems for full containment conditions.

The compact architecture of free-floating duckweed and simple method of aseptic cultivation on low-cost nutrient solutions demonstrate the possibility for economically feasible whole duckweed-based production cycles (from growing plants to harvesting biomass with accumulated target products) in fully controlled, aseptic, and biosafe conditions even in large-scale industrial processes. Potentially, these advantages will allow faster, simpler, and cheaper current Good Manufacturing Practice (cGMP) certification of duckweed-based recombinant product production sites compared to terrestrial plant-based systems. Moreover, Nguyen and coauthors (2005) previously proposed engineering of duckweed auxotrophs as a promising approach to meet regulatory requirements (MacDonald et al., 2005) and prevent the potential release of biomass into the environment or a commodity stream for which it is not intended.

Further development of the tools for plant-made pharmaceutical technology is necessary to expand the availability of quality medicines, thereby diversifying the strategies available to address global pandemics and endemic diseases (Tusé et al., 2020). Transient expression in duckweed could be a valuable tool for research and development of duckweed-based technologies for biopharmaceutical production at proof-of-concept stages. To be successful, research and development of new biotechnological pharmaceutical products should be based on cheap and high-throughput methods to facilitate proof-of-concept testing and later large-scale production. The transient expression approach allows research and development to bypass the labor- and time-consuming generation of stably transformed plants, and provides an opportunity to optimize the expression vectors, to investigate potential toxicity of target products for the host cells, to test possible purification schemes, and to check the chemical structure and biological properties of the target product. Therefore, duckweed is a promising host organism for transient expression systems, combining high-yield expression with fully contained and controlled cultivation, which are critical requirements for the pharmaceutical manufacturing industry and environmental biosafety.

## Data Availability

The original contributions presented in the study are included in the article/Supplementary Material, further inquiries can be directed to the corresponding authors.
