# Supplementary material for: Robust Agrobacterium-Mediated Transient Expression in Two Duckweed Species (Lemnaceae) Directed by Non-replicating, Replicating, and Cell-to-Cell Spreading Vectors
Source: Front Bioeng Biotechnol. 2021 Nov 4;9:5. doi: 10.3389/fbioe.2021.761073 (PMC8600122; doi:10.3389/fbioe.2021.761073)
Supplement: Supplementary file 1 [file DataSheet1.docx]

Supplementary Material

# Supplementary Methods

**Supplementary Method 1**

**Reference plants.** *Lactuca sativa* and *Nicotiana benthamiana* were used reference plants in this study*.* Fresh *Lactuca sativa* plants cut from their roots were purchased from a local supermarket. Several *L. sativa* varieties were purchased and tested in preliminary experiments to select the most suitable material for reproducible transient expression. Based on the results, Chinese variety "生菜Shēngcài" was chosen for further experiments.

Plantlets of *N. benthamiana* were germinated from seeds using Klasmann-Deilmann peat moss Rec. #422, watered with 0.1x SH macronutrients, and grown for 5 weeks in a climate chamber at 24 ± 1 °C with a photon flux density of 150–250 μmol m^−2^ s^−1^ provided by Cool White fluorescent bulbs in a 16-h light/8-h dark cycle.

**Supplementary Method 2**

**Maintaining reference plant materials after Agroinfiltration.** Following infiltration with *Agrobacterium*, the *L. sativa* and *N. benthamiana* plants were transferred to transparent 6-l polypropylene boxes filled with 1–2 cm of tap water at the bottom and fixed in a vertical position. Boxes were covered with transparent polyethylene wrap and kept in darkness at 22 °C in the climate chamber for the first 24 h. The incubation was followed by incubation in a 12-h/12-h light/dark regime with the temperature set at 18 °C for the light period and 16 °C for the dark period. To prevent microbial growth, the tap water in the boxes was changed every day.

**Supplementary Method 3**

**Observation and photography of GFP fluorescence and sampling of plant material for evaluation of GFP accumulation.** Visual monitoring of reporter protein accumulation, performed twice per day to choose the appropriate time for protein extraction, and photo-documenting of specific fluorescence *in planta* were performed in darkness using a 100 mW, 488 nm LED laser light source for plants infiltrated with *Agrobacterium* carrying the SPLCV-GFP, 35S-GFP, and Ubi-GFP vectors, and a 500 mW, 405 nm LED laser light source for plants infiltrated with *Agrobacterium* carrying the 35S-GFP-p19 and PVX-GFP vectors. The fluorescence induced by the 405 nm LED laser was observed using common industrial protective eyepieces (yellow color with "UV400-stop" marking), whereas the 488 nm LED laser-induced fluorescence was monitored using modified welding eyepieces, where the light screen was replaced with two 520 nm light filters (50x50 mm). For photo-documenting, the same light sources were used in combination with 450 nm light filters (for 405 nm laser) and 520 nm light filters (for 488 nm laser), fixed to photo-camera objectives. Alternatively, plants were photographed using artificial white light from a daylight fluorescent lamp. Photo-documenting was performed using Canon PowerShot A1200 or Canon EOS M200 cameras. Images taken in RAW format were then processed and converted to TIF or JPEG format using Canon Digital Photo Professional 4 software.

For measurement of the accumulated GFP, about 10 g of fresh *L. sativa* or *N. benthamiana* tissues completely involved in maximal transient expression, as confirmed by visual observation, was used for protein extraction. The *L. sativa* leaves inoculated with the Ubi-GFP, 35S-GFP, or SPLCV-GFP vectors were collected at 2–3 days post infiltration (dpi). Sampling of *N. benthamiana* plants infiltrated with *Agrobacterium* suspension carrying the non-replicating 35S-GFP-p19 vector was performed at 3–4 dpi. For protein extraction from plant material inoculated with the non-replicating vectors, the central veins of each leaf were excluded.

The samples of *N. benthamiana* plants inoculated with the replicating and cell-to-cell spreading vector PVX-GFP were taken from whole leaves, including central veins, between 11 and 14 dpi.

**Supplementary Method 4**

**Expression of sGFP and mGFP5 in *E. coli* and preparation of standards.** To prepare protein standards for quantification of the GFP expressed in plants, the two GFP variants used as reporters in plants (sGFP and mGFP5) were expressed in *E. coli*. The sequence of the *mGFP5-6His* gene was amplified by PCR from the DNA of the SPCLV-GFP vector using gene-specific primers 5′-GACCATGGTGAGCAAGGGCGAGG-3` and 5`-GAGAAGCTTAGTGGTGGTGGTGGTGGTGC-3′. The amplification product was digested with *Nco*I and *Hind*III and cloned into the pQE-60 vector (Qiagen). Similarly, the sequence of the *sGFP* gene was amplified from 35S-GFP-p19 vector with primers 5′-GACCATGGTGAGCAAGGGCGAGG-3′ and 5′-GAGAGATCTCTTGTACAGCTCGTCC-3′. The resulting DNA fragment was digested with *Nco*I and *Bgl*II and cloned into the pQE-60 vector (Qiagen). The obtained recombinant plasmids, pQE-60-mGFP5-6His and pQE-60-sGFP-6His, were used for expression and purification of GFP from *E. coli* strain XL-Blue1.

To prevent formation of inclusion bodies and to maximize correct folding of GFP in soluble form, bacteria were cultivated at lower temperature (30 °C instead of 37 °C), using minimal nutrition medium (Studier, 2005) and a minimal concentration of isopropyl β-D-thiogalactoside (IPTG) inducer (0.1 mM). After cultivation for 24 h, bacterial biomass was lysed overnight at 4 °C with 8 M urea and 0.1 mM phenylmethylsulfonyl fluoride (PMSF), using an end-over-end shaker. After lysis, the suspensions were centrifuged for 20 min at 12,000 *g* and 4 °C and the supernatants were used for further purification of the GFPs.

The produced GFPs were further purified from *E. coli* lysates using Ni-Charged MagBeads (GenScript, Cat. No. L00295) according to the manufacturer’s protocol for batch purification. Elution of GFP from magnetic beads was performed using 0.05 M phosphate buffer solution supplemented with 0.3 M NaCl and 0.1 mM PMSF, pH 4.5, followed by pH neutralization using 1 M phosphate buffer, pH 8.0. The purity of GFPs in the eluates was evaluated by sodium dodecyl sulfate polyacrylamide gel electrophoresis (SDS-PAGE) according to the BIO-RAD Mini-PROTEAN Tetra Cell Instruction Manual. The concentrations of purified GFPs were measured by micro-biuret method (Itzhaki and Gill, 1964).

# Supplementary Data

**Dynamics of GFP expression in reference plants**. The first GFP fluorescence visible to the naked eye in *L. sativa* plants inoculated with the Ubi-GFP, 35S-GFP, and SPLCV-GFP vectors was detectable at 2 dpi. A further increase in GFP fluorescence was observed over the next one or two days. The dramatic withering/necrosis of the plants usually occurred at 3–5 dpi (**Supplementary Figure 1A – F)**. The brightness of GFP fluorescence in *L. sativa* plants transfected with the non-replicating vector 35S-GFP was not as strong as for the replicating vector SPLCV-GFP, but the involvement of the plant tissue in expression was the same as for the SPLCV-GFP vector. Compared to the plants with 35S-GFP vector, the pattern of GFP expression from the vector Ubi-GFP was significantly different, with dramatically decreased brightness of GFP fluorescence and sporadic distribution of fluorescent spots.

For *N. benthamiana* plants inoculated with the non-replicating vector 35S-GFP-p19, the first signs of expression visible to the naked eye were detected at 2 dpi. A further increase in the brightness of GFP fluorescence was observed during the next two or three days, when the complete involvement of the plant leaves, excluding veins, in GFP expression, was observed (**Supplementary Figure 1G**). From 5–6 dpi, the brightness of GFP fluorescence usually started to decrease and completely disappeared at 10–12 dpi.

For the replicating and cell-to-cell spreading vector PVX-GFP, the first signs of specific GFP fluorescence were detected at 3 dpi, with a further increase in GFP fluorescence observed during the next four to six days. During this time, the complete involvement of the plants, including the stem, petioles and leaf veins, in GFP expression was observed with fluorescence brightness usually much higher compared to the 35S-GFP-p19 vector. Representative images of *N. benthamiana* transfected with the 35S-GFP-p19 and the PVX-GFP vectors are provided in **Supplementary Figure 1H**).

Signs of withering in the *N. benthamiana* plants usually started to appear at 14–16 dpi.

# Supplementary Figures


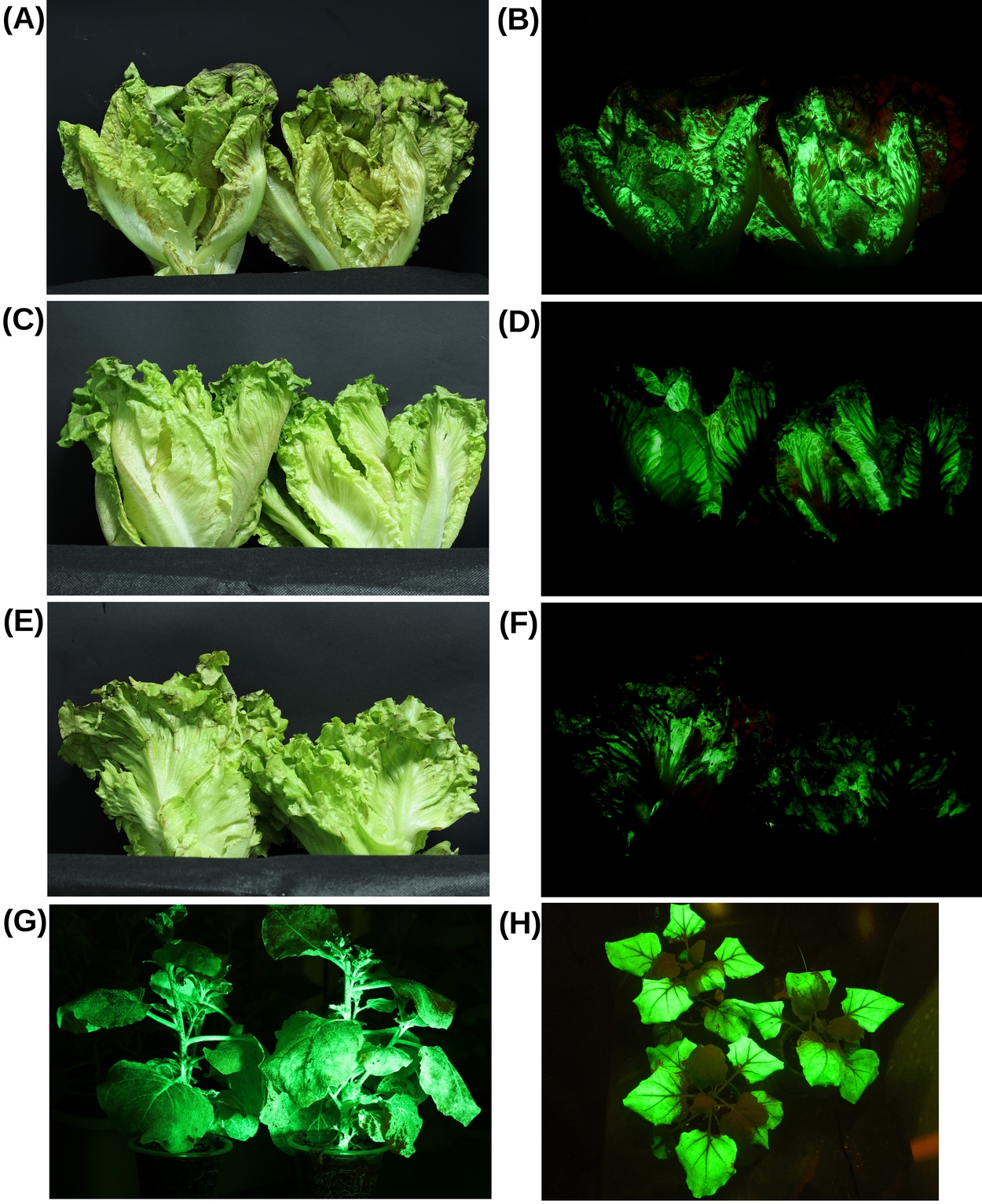


**Supplementary Figure 1.** Representative photos of reference plants expressing GFP. **(A), (B)** *L. sativa* infiltrated with *Agrobacterium* bearing the SPLCV-GFP vector, 3 dpi. **(C), (D)** *L. sativa* infiltrated with *Agrobacterium* bearing the 35S-GFP vector, 3 dpi. **(E), (F)** *L. sativa* infiltrated with *Agrobacterium* bearing the Ubi-GFP vector, 3 dpi. **(G)** *N. benthamiana* infiltrated with *Agrobacterium* bearing the PVX-GFP vector, 12 dpi. **(H)** *N. benthamiana* infiltrated with *Agrobacterium* bearing the 35S-GFP-p19 vector, 4 dpi. **(A)**, **(C)**, **(E)** Images taken under artificial white light. **(B)**, **(D)**, **(F)** Images taken under monochrome excitation light (488 nm) through a long wave pass light filter (520 nm). **(G)**, **(H)** Images taken under monochrome excitation light (400 nm) through long wave pass light filter (450 nm).


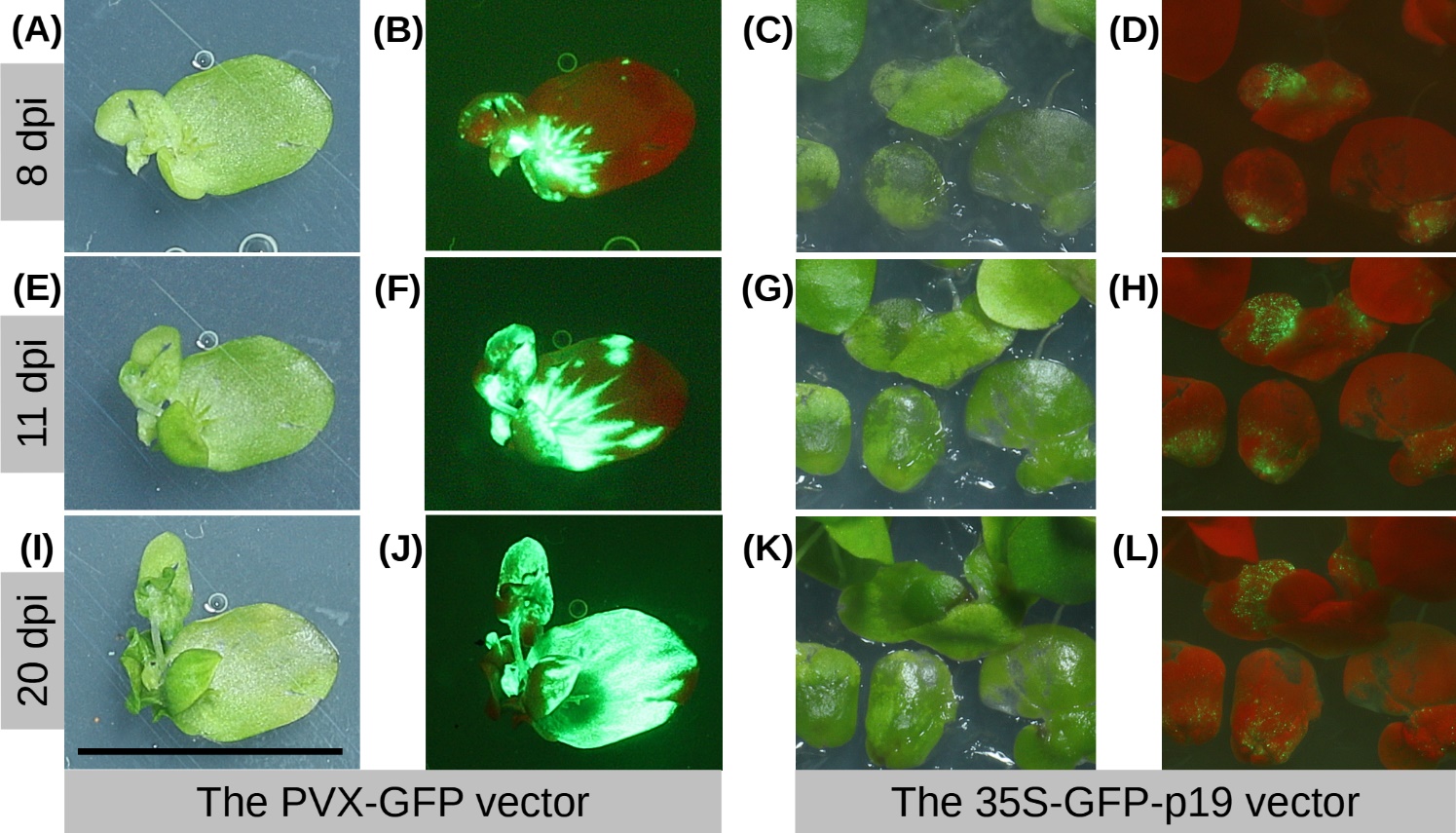


**Supplementary Figure 2.** Time-course of GFP fluorescence dynamics on the surface of *S. polyrhiza* fronds infiltrated with *Agrobacterium* bearing the PVX-GFP and 35S-GFP-p19 vectors. Representative photos of the same *S. polyrhiza* fronds expressing GFP at **(A)**–**(D)** 8 dpi; **(E)**–**(H)** 11 dpi; **(I)**–**(L)** 20 dpi. **(A)**, **(B)**, **(E)**, **(F)**, **(I)**, **(J)** Fronds infiltrated with *Agrobacterium* bearing the PVX-GFP vector. **(C)**, **(D)**, **(G)**, **(H)**, **(K)**, **(L)** Fronds infiltrated with *Agrobacterium* bearing the 35S-GFP-p19 vector. **(A)**, **(C)**, **(E)**, **(G)**, **(I)**, **(K)** Images taken under artificial white light. **(B)**, **(D)**, **(F)**, **(H)**, **(J)**, **(L)** Images taken under monochrome excitation light (400 nm) through long wave pass light filter (450 nm). Bar = 1 cm.


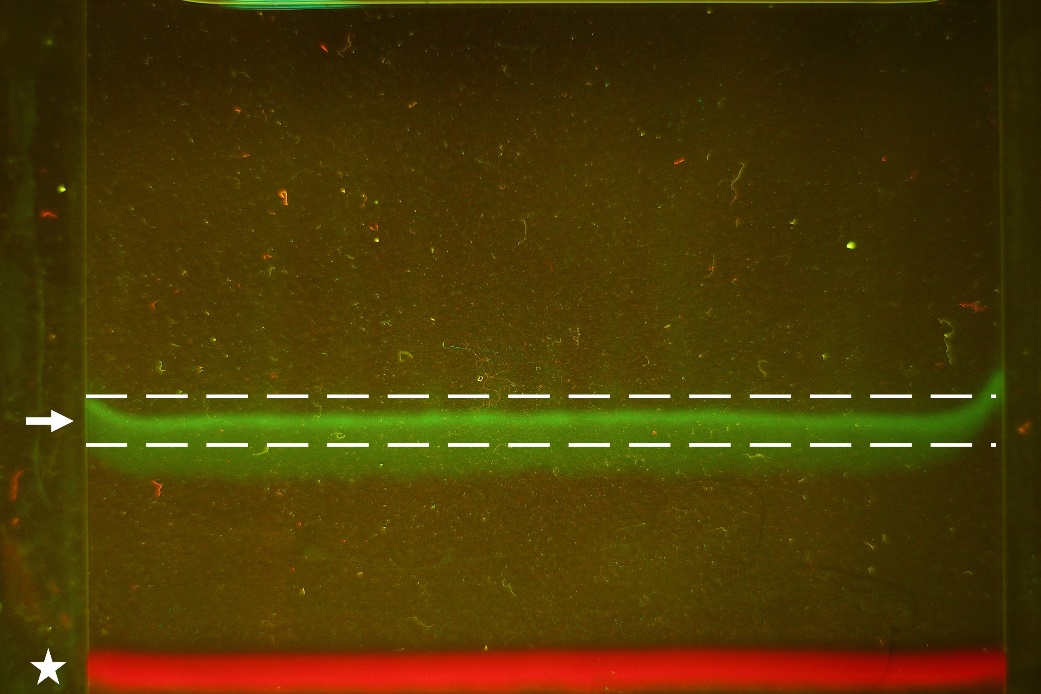


**Supplementary Figure 3**. SDS-PAGE gel after separation of crude protein extract from *L. punctata* infiltrated with *Agrobacterium* bearing the SPLCV-GFP vector (representative image, 25 dpi). the white arrow indicates the specific fluorescent band for GFP. The two white dashed lines delimit the region of the gel to be cut for GFP elution and downstream measurement of fluorescence in the eluate. The white star indicates the gel area containing fluorescent pigments, mainly chlorophyll (red fluorescence). The image was taken under monochrome excitation light (488 nm) through a long wave pass light filter (520 nm).


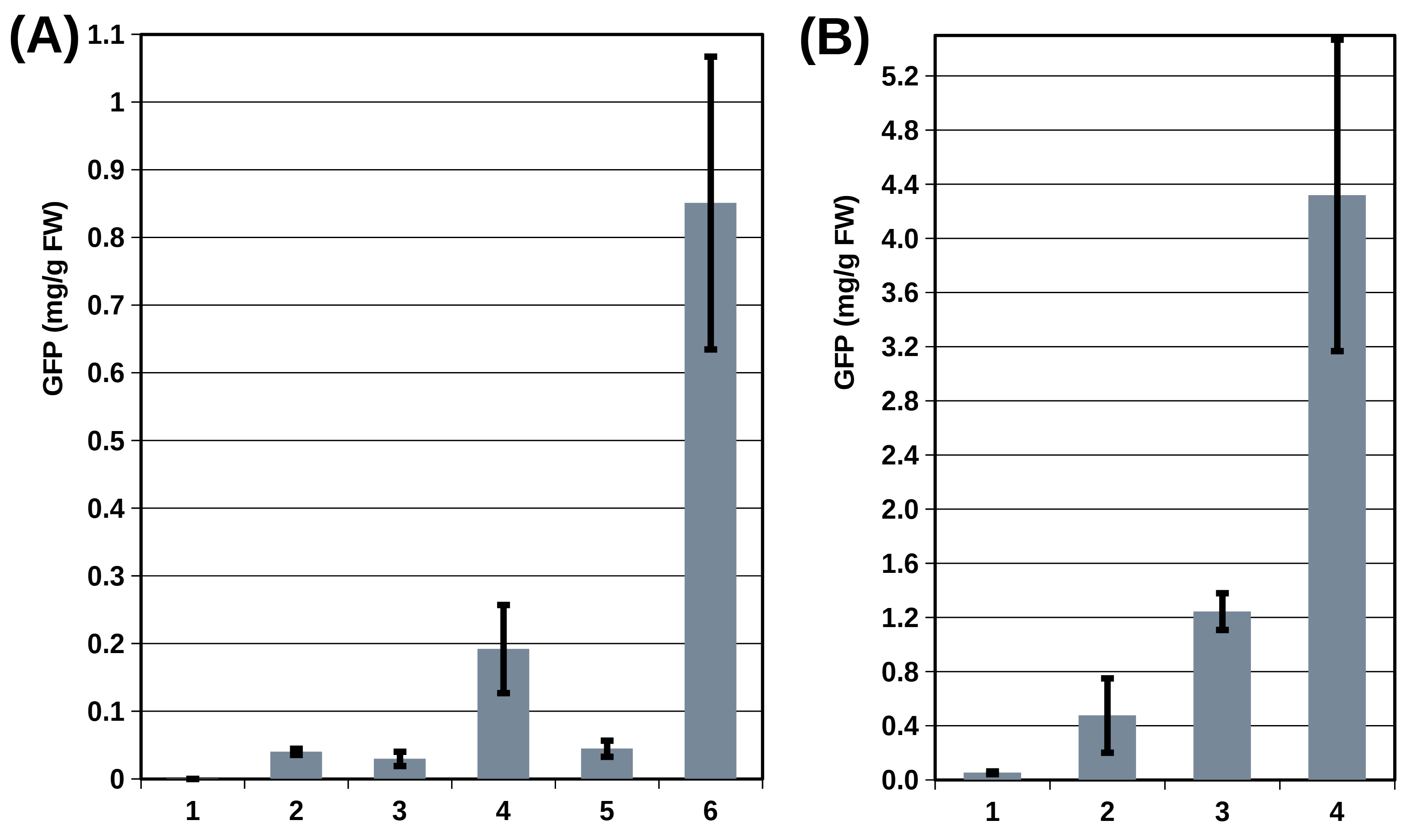


**Supplementary Figure 4.** GFP accumulation in plants infiltrated with *Agrobacterium* bearing different vectors (plantwith vector): **(A)1** *L. punctata* with the Ubi-GFP vector (no accumulation), 10–12 dpi; **(A)2** *L. sativa* with the Ubi-GFP vector, 2–3 dpi; **(A)3** *L. punctata* with the 35S-GFP vector, 10–12 dpi; **(A)4** *L. sativa* with the 35S-GFP vector, 2–3 dpi; **(A)5** *L. punctata* with the SPLCV-GFP vector, 25–30 dpi; **(A)6** *L. sativa* with the SPLCV-GFP vector, 2–3 dpi; **(B)1** *S. polyrhiza* with the 35S-GFP-p19 vector, 10–12 dpi; **(B)2** *N. benthamiana* with the 35S-GFP-p19 vector, 3–4 dpi; **(B)3** *S. polyrhiza* with the PVX-GFP vector, 20–25 dpi; **(B)4** *N. benthamiana* with the PVX-GFP vector, 11–14 dpi. Error bars indicate doubled standard deviations (*n*=3).

**References**

1. Studier, F.W. (2005) Protein production by auto-induction in high-density shaking cultures. *Protein Expression and Purification*, **41**, 207–234.

2. Itzhaki, R.F. and Gill, D.M. (1964) A micro-biuret method for estimating proteins. *Analytical Biochemistry*, **9**, 401–410.
